# Identification of a Disease-Associated Network of Intestinal Immune Cells in Treatment-Naive Inflammatory Bowel Disease

**DOI:** 10.3389/fimmu.2022.893803

**Published:** 2022-06-23

**Authors:** Vincent van Unen, Laura F. Ouboter, Na Li, Mette Schreurs, Tamim Abdelaal, Yvonne Kooy-Winkelaar, Guillaume Beyrend, Thomas Höllt, P. W. Jeroen Maljaars, M. Luisa Mearin, Ahmed Mahfouz, Anne M. C. Witte, Cornelis H. M. Clemens, Sunje Abraham, Johanna C. Escher, Boudewijn P. F. Lelieveldt, M. Fernanda Pascutti, Andrea E. van der Meulen – de Jong, Frits Koning

**Affiliations:** ^1^ Department of Immunology, Leiden University Medical Center, Leiden, Netherlands; ^2^ Institute for Immunity, Transplantation, and Infection, Stanford University School of Medicine, Stanford, CA, United States; ^3^ Department of Gastroenterology, Leiden University Medical Center, Leiden, Netherlands; ^4^ Delft Bioinformatics Lab, Delft University of Technology, Delft, Netherlands; ^5^ Computer Graphics and Visualization, Delft University of Technology, Delft, Netherlands; ^6^ Department of Pediatrics, Leiden University Medical Center, Leiden, Netherlands; ^7^ Department of Human Genetics, Leiden University Medical Center, Leiden, Netherlands; ^8^ Leiden Computational Biology Center, Leiden University Medical Center, Leiden, Netherlands; ^9^ Department of Gastroenterology, Alrijne Hospital, Leiden, Netherlands; ^10^ Department of Pediatric Gastroenterology, Erasmus University Medical Center, Rotterdam, Netherlands; ^11^ Pattern Recognition and Bioinformatics Group, Delft University of Technology, Delft, Netherlands; ^12^ Department of The Division of Imaging Processing (LKEB) Radiology, Leiden University Medical Center, Leiden, Netherlands

**Keywords:** inflammatory bowel diseases, Crohn’s disease, ulcerative colitis, mass cytometry, single-cell analysis, intestinal immune cell network, mucosal immunology, CyTOF

## Abstract

Chronic intestinal inflammation underlies inflammatory bowel disease (IBD). Previous studies indicated alterations in the cellular immune system; however, it has been challenging to interrogate the role of all immune cell subsets simultaneously. Therefore, we aimed to identify immune cell types associated with inflammation in IBD using high-dimensional mass cytometry. We analyzed 188 intestinal biopsies and paired blood samples of newly-diagnosed, treatment-naive patients (*n*=42) and controls (*n*=26) in two independent cohorts. We applied mass cytometry (36-antibody panel) to resolve single cells and analyzed the data with unbiased Hierarchical-SNE. In addition, imaging-mass cytometry (IMC) was performed to reveal the spatial distribution of the immune subsets in the tissue. We identified 44 distinct immune subsets. Correlation network analysis identified a network of inflammation-associated subsets, including HLA-DR^+^CD38^+^ EM CD4^+^ T cells, T regulatory-like cells, PD1^+^ EM CD8^+^ T cells, neutrophils, CD27^+^ TCRγδ cells and NK cells. All disease-associated subsets were validated in a second cohort. This network was abundant in a subset of patients, independent of IBD subtype, severity or intestinal location. Putative disease-associated CD4^+^ T cells were detectable in blood. Finally, imaging-mass cytometry revealed the spatial colocalization of neutrophils, memory CD4^+^ T cells and myeloid cells in the inflamed intestine. Our study indicates that a cellular network of both innate and adaptive immune cells colocalizes in inflamed biopsies from a subset of patients. These results contribute to dissecting disease heterogeneity and may guide the development of targeted therapeutics in IBD.

## Introduction

Inflammatory bowel disease (IBD) is an expanding global health problem characterized by chronic inflammation of the intestine. The incidence and prevalence of IBD are rising worldwide (1–3), and the etiology is multifactorial that depends upon genetics, dysregulated immune responses, and environmental factors, including the intestinal microbiota (4, 5). The primary forms are Crohn’s disease (CD) and ulcerative colitis (UC). Endoscopic evaluation is a hallmark for diagnosis and management of IBD, which is, however, invasive, costly, and time-consuming. In addition, the treatment for IBD is usually lifelong and requires expensive pharmacotherapy (6, 7), yet remission is challenging to maintain (8), and responses are variable and unpredictable (9). Therefore, improved classification of IBD is highly desired, as are biomarkers to predict response to treatment, novel diagnostics and therapeutic approaches.

It has been shown that IBD**-**associated inflammation can result from aberrant immune responses against the microbiota by dendritic cells (10) and CD4^+^ T cells (10–12). While many studies (13) have investigated the role of immune subsets in IBD, it has been challenging to interrogate the role of all immune subsets in an integrated manner. High**-**dimensional mass cytometry allows the analysis of >40 cellular markers simultaneously (14). With such an approach, we have previously observed a high number of diverse immune cell types in the intestine and both inter- and intraindividual variation (15). Therefore, in the current study, we applied mass cytometry to analyze the composition of immune cells in biopsies and paired peripheral blood mononuclear cell (PBMC) samples of treatment-naïve IBD patients and controls, aged 10-40 years old. Here, we have comprehensively delineated a network of multiple innate and adaptive immune cell types associated with inflammation in the intestine that was highly distinct from unaffected controls in two independent cohorts. The composition of IBD-associated immune subsets was prominent in both inflamed colon and inflamed ileum and did not separate CD and UC. Comprehensive profiling of the cellular immune system in IBD is a valuable resource to improve patient classification and might aid in the development of personalized strategies.

## Materials and Methods

### Human Samples

Patients (primary cohort: IBD, *n*=23; control, *n*=11 and validation cohort: IBD, *n*=19; control, *n*=15) eligible for inclusion were aged 10-40 years, and were undergoing diagnostic ileocolonoscopy due to clinical suspicion of newly diagnosed IBD. All patients were naive for any IBD medication (e.g., corticosteroids, 5-ASA or any immunosuppressive therapy). The patient characteristics are shown in **Table S2**. Samples from the rectum, colon and ileum, both from inflamed and noninflamed mucosa if available, were collected. If no abnormalities were seen at colonoscopy, these individuals were included as controls. No patients with microscopic colitis were present in the current cohort. CD patients were classified according to the Montreal classification (16). The inflamed segment of each patient was classified according to the SES-CD, as inactive (0-2), mild (3-6), or moderate-severe (≥7) (17). UC patients were categorized according to the extent of disease (rectum, S1; left-sided, S2, or pancolitis, S3) and severity of disease according to the endoscopic Mayo score. Three patients were diagnosed as IBDU (unclassified), and were included in the UC group.

### Isolation of Cells From Intestinal and PBMC Samples

The isolation of intestinal biopsies and PBMC samples were isolated as previously described (15). Briefly, intestinal biopsies (*n*=188) were collected in HBSS (Sigma-Aldrich) medium after endoscopy. Cells from the epithelium were isolated by treatment with 10 mL of HBSS containing 1 mM EDTA (Merck) under rotation for 1.5 hours at 37°C. To obtain cells from the lamina propria, we washed the biopsies with PBS containing 0.5% fetal calf serum (FCS) and incubated with 5 mL of a collagenase mix containing IMDM culture medium (Lonza) with 20% FCS, 1,000 U/mL collagenase IV (Worthington), and 10 mg/mL DNAseI grade II (Roche Diagnostics) for 1.5 hours at 37°C. Cells isolated from both the epithelium and lamina propria were then together filtered through a 70 µm cell strainer and centrifuged in 0.5% FCS/PBS.

Peripheral blood samples (*n*=68) were isolated from up to 5 mL freshly drawn heparin anticoagulated blood using Ficoll-Paque™ density-gradient centrifugation. Isolated cells were washed with PBS, counted and 2 million cells were stored in 20% FCS/RPMI and stored at 4°C until antibody staining.

### Antibody Staining and Data Acquisition

The validated mass cytometry antibody panel composition is similar as applied before (18). Heavy metal isotope-tagged monoclonal antibodies are listed in **Table S1**. Procedures for conjugation of the antibodies, mass cytometry antibody staining, and data acquisition were performed as described (15). Briefly, purified antibodies were conjugated with heavy metal reporters in-house using the MaxPar X8 Antibody Labeling Kit (Fluidigm) according to the manufacturer’s instructions. Directly after isolation of single-cells, we resuspended cells in cell staining buffer (CSM; 1xPBS with 0.5% bovine serum albumin and 0.02% sodium azide, Fluidigm Sciences) and incubated with 1 mL of 1:500 diluted 500 mM rhodium DNA intercalator (Fluidigm Sciences) for 15 min to stain dead cells at room temperature (rT). Cells were washed with CSM and surface stained for 45 min at rT with the mixture listed in **Table S1**. After staining, cells were washed twice with CSM and then resuspended in 1 mL of 1:1,000 diluted 125 mM iridium DNA intercalator (Fluidigm Sciences) in Fix and Perm Buffer (PBS with 1.6% paraformaldehyde, Fluidigm Sciences) to discriminate single cells. Cells were stored overnight at 4°C up to 48 hours. Finally, cells were washed twice in CSM and once in distilled water at rT. Prior to data acquisition, cell pellets were diluted in distilled water containing 1:10 diluted EQ Four Element Calibration Beads (Fluidigm Sciences) to the concentration of 0.4*10^6^ cells/mL to achieve an acquisition rate of 500 events/s on the CyTOF 2™ and Helios mass cytometer (Fluidigm Sciences). CyTOF data were acquired and analyzed on-the-fly, using dual-count mode and noise-reduction on. All other settings were either default settings or optimized with tuning solution, as instructed by Fluidigm Sciences. After data acquisition, the mass bead signal was used to normalize the short-term signal fluctuations with the reference EQ passport P13H2302 during the course of each experiment.

### Imaging Mass Cytometry

Inflamed biopsies from UC patients (*n*=4) and noninflamed biopsies from controls (*n*=4) were used for IMC. Preparation and staining of slides were performed as previously described (19). Briefly, sections were cut from snap-frozen tissues at 5 µm and mounted on silane-coated glass slides, fixed with 1% PFA for 5 min at rT, followed by 100% cold methanol for 5 min at -20°C. Sections were subsequently blocked for 30 min in Superblock (Thermo Superblock Solutions) at rT and then incubated with the antibody mix overnight at 4°C (**Table S3**). The following day, sections were stained with intercalator-Ir (dilution 1:400, Fluidigm) and stored at 4°C until ablation. Acquisition was performed on a Helios time-of-flight mass cytometer coupled to a Hyperion Imaging System (Fluidigm). After flushing the chamber with helium, tissues were ablated by a UV-laser spot-by-spot at a resolution of 1 µm with a frequency of 200 Hz. Prior to sample ablation, the instrument was tuned according to the manufacturer’s instructions, using the 3-Element Full Coverage Tuning Slide (Fluidigm). Regions of interest (ROIs) with a maximum of 1,000 µm × 1,000 µm were selected. We ablated 2-8 ROIs for each tissue section. All raw data were analyzed for marker intensity based on the maximum signal threshold, defined at the 98th percentile of all pixels in a single ROI using the Fluidigm MCD™ viewer (v1.0.560.2). To distinguish the signal from background, we used the Fluidigm MCD™ viewer to visualize our data, and adjusted the Threshold Min and Max values for each marker individually to eliminate background.

### Data Analysis

**Figure 1:**
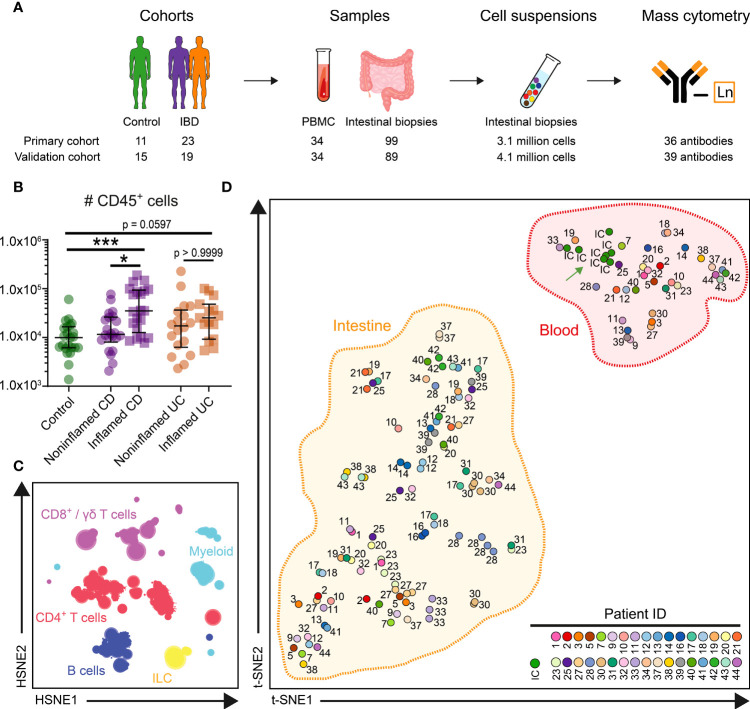
HSNE identifies major immune lineages in blood and intestine. **(A)** Experimental approach **(B)** Graph showing absolute number of CD45^+^ immune cells acquired from 99 intestinal biopsies of the primary cohort. Bars indicate the median with interquartile range. ^*^p≤0.05, ^***^p≤0.001, Kruskal Wallis test for multiple groups. **(C)** Overview HSNE embedding of 7.7 million immune cells. Color represents the major lineages. **(D)** t-SNE analysis (% of CD45^+^ cells) of clustered cells for 133 samples and 7 internal control (IC) replicate samples. Every dot represents a sample, and the color and number display 34 individuals and the IC. t-SNE analysis (% of CD45+ cells) of clustered cells for 133 samples and 7 internal control (IC) replicate (green arrow) samples.

**Figure 2:**
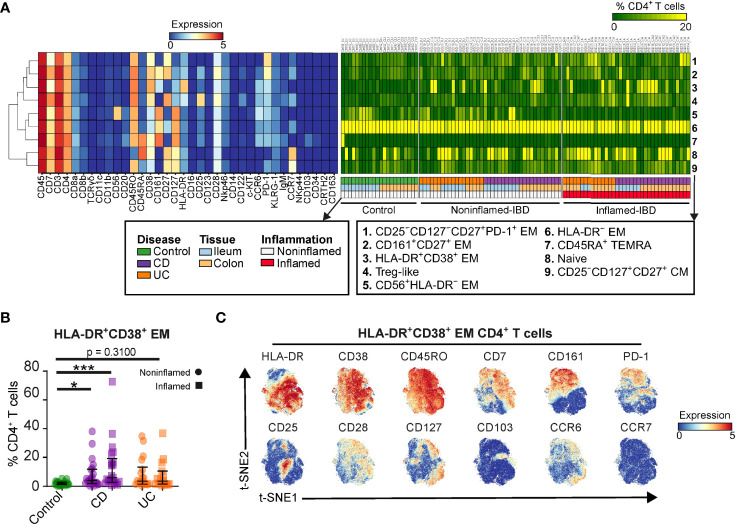
The CD4^+^ T cell lineage dissected into distinct cell subsets. **(A)** Heatmaps showing characteristics of 9 CD4^+^ T cell subsets (marker expressions; rainbow scale), cell subset composition of intestinal samples (cell percentages; green-to-yellow scale), clinical metadata and hierarchical clustering of marker expression profiles. **(B)** Graph showing cell frequency of HLA-DR^+^CD38^+^ EM CD4^+^ T cell cluster for 99 intestinal biopsies of the primary cohort. Bars indicate the median with interquartile range. ^*^p≤0.05, ^***^p≤0.001, Kruskal Wallis test for multiple groups. **(C)** t-SNE embedding showing 61,930 cells colored for the expression values of the indicated markers.

**Figure 3:**
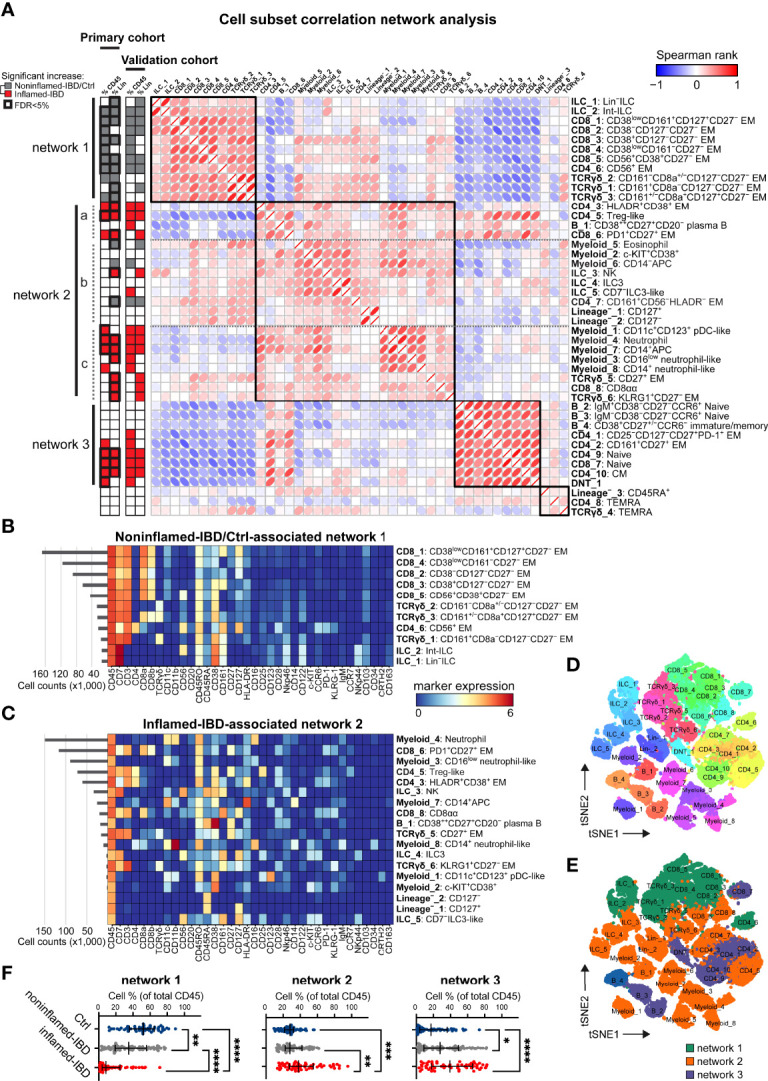
Integrated immune system analysis identifies IBD-associated cellular networks. **(A)** Network representation of correlations (Spearman rank) between 44 immune subsets. Hierarchical clustering of correlations indicated by four black squares. Statistics bar (left panel) indicating significant (p<0.05) differences in cell frequencies comparing inflamed-IBD (in red) samples with noninflamed-IBD/control samples (in grey) as determined with independent t-tests stratified for the primary and the validation cohort. False discovery rate <5% highlighted with bold outline for the primary cohort. Heatmaps showing maker expressions of relevant cell subsets from **(B)** network 1 and **(C)** network 2 as defined in panel **(A)** t-SNE maps of immune subsets (each downsampled to 1,000 cells) from the three networks and colored for **(D)** immune subset and **(E)** corresponding network. **(F)** Graphs showing the collective cell subset frequencies (as % of total CD45^+^ cells) of network 1, network 2 and network 3, for 53 control, 76 noninflamed-IBD and 59 inflamed-IBD intestinal samples from the primary and the validation cohorts combined. Bars indicate the median with interquartile range. ^*^p≤0.05, ^**^p≤0.01, ^***^p≤0.001, ^****^p≤0.0001, Kruskal Wallis test for multiple groups.

**Figure 4:**
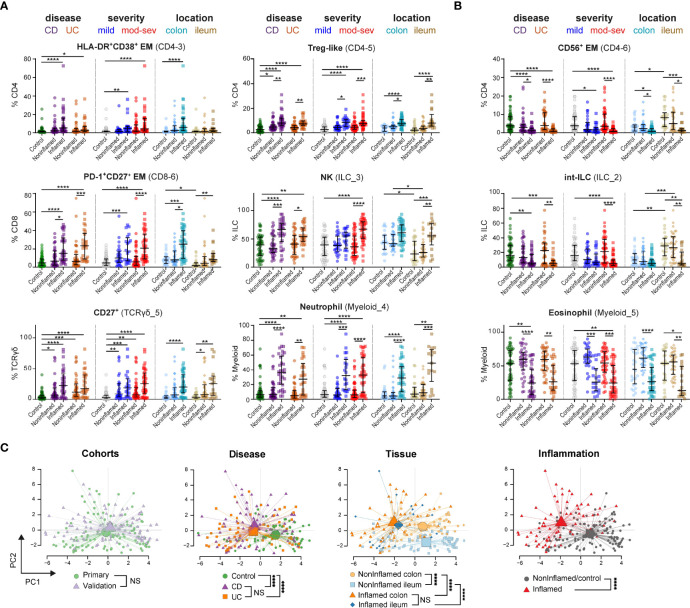
Immune subset signatures stratified for disease subtype, severity and intestinal location. Graphs show frequencies (as % of immune lineage) of indicated cell subsets from **(A)** network 2 and **(B)** network 1 in each intestinal biopsy from 53 controls, 76 noninflamed-IBD samples and 59 inflamed-IBD samples from the primary and validation cohorts combined, stratified for disease subtype (CD, Crohn’s disease; UC, ulcerative colitis), disease severity (mild and mod-sev, moderate-to-severe) and intestinal location. Bars indicate the median with interquartile range. ^*^p≤0.05, ^**^p≤0.01, ^***^p≤0.001, ^****^p≤0.0001, Kruskal-Wallis test for multiple groups. **(C)** PCA (% of CD45^+^ cells) of 44 immune cell subsets for 188 intestinal samples. Every dot is an intestinal sample and colors show the cohort, disease subtype, tissue location and inflammation state. Lines connect samples to the centroid of each group. ****p≤0.0001, Hotelling’s two-sample T2-test for PCA-reduced sample groups. NS indicates not significant.

Cytobank was used to gate out beads, and we discriminated live, single CD45^+^ immune cells with DNA stain and event length. We included a standardized blood sample from the same healthy individual that was drawn at a single time point and cryopreserved in multiple tubes as an internal control. HSNE analyses were performed as described (20) using the default settings in the Cytosplore software (21) application. The blood and intestinal samples were analyzed together with HSNE in **Figure 1** and the intestinal samples alone in a separate HSNE analysis (**Figures 2**–**4**). The publicly available single-cell RNA-seq dataset (22) was analyzed with the R-package ‘Seurat’ (23). The R-package ‘CytoFast’ (24) was used to calculate the differential abundance of cell clusters, and the R-package ‘corrplot’ (25) was used to calculate the correlation networks of cell frequencies of the identified cell subsets. The standard pre-processing was applied as for the t-SNE sample analysis prior to the correlation network analysis. Finally, we used the LDA classification approach (26) to identify the primary cohort-defined cell subsets in the validation cohort samples and the intestinal samples-defined clusters in the peripheral blood samples. We trained the LDA classifier using the intestinal cell clusters of the primary cohort and was used to automatically assign cells from the validation cohort to the most similar primary cohort-defined cell cluster. For peripheral blood, the trained LDA classifier was used to predict similar intestinal cell clusters for blood cells, using a rejection option with two different minimum posterior probability thresholds of 1 and 0.95, to produce two levels of prediction confidence of 100% and 95%, respectively. The primary and validation cohort were pooled in an integrated analysis to reveal associations of cell subsets when stratified for IBD subtype, severity and intestinal location. PCA of the samples for immune cluster frequencies was performed after the same standard pre-processing was applied as for the t-SNE sample analysis. The centroid for each patient group is plotted in the PCA.

### Statistical Analysis

Independent *t*-tests were performed comparing IBD-inflamed specimens with IBD-noninflamed/control specimens with resulting p-values adjusted for multiple hypothesis testing (FDR<5%) in the correlation network analysis (**Figure 3**). The cell clusters of interest were plotted in bar graphs stratified for the different subtypes of IBD, and inflamed or noninflamed mucosa, and disease severity groups and intestinal locations (**Figure 4**). Here, the Kruskal-Wallis test with Dunn’s test for multiple comparisons was performed (Graphpad V.9) and the cell cluster frequencies were plotted for the individual samples along with the median.

### Study Approval

This study was approved by the Medical Ethical Committee of the Leiden University Medical Center (LUMC) (protocol P15.193). All samples were obtained after informed consent, medical ethical commission approval, in accordance with the local ethical guidelines of the LUMC, Erasmus Medical Center and Alrijne hospital, and in accordance with the Declaration of Helsinki.

## Results

### Stable Intraindividual Immune Profiles in the Intestine

We designed a 36-antibody panel to obtain an overview of the heterogeneity of the innate and adaptive immune system (**Table S1**). We incorporated markers that distinguished the six major immune lineages (CD4^+^, CD8^+^, and TCRγδ T cells, innate lymphoid cells [including NK cells and CD127^+^ helper-ILC], B and myeloid cells), markers indicative of cell activation, maturation, developmental stages, and responsiveness to humoral factors. All antibodies have been extensively validated (18). With this panel, single-cell suspensions derived from biological specimens were analyzed. In addition, a validation cohort was analyzed with an optimized 39-antibody panel (**Table S1**).

Intestinal samples, both from noninflamed and inflamed mucosa, were collected from treatment-naïve patients undergoing diagnostic ileocolonoscopy. IBD patients were classified according to the Montreal classification (16, 27). Individuals without luminal disease (endoscopically and pathological report) were assigned to the control group. The samples from the primary cohort include ileum biopsies (*n*=41), colon biopsies (*n*=58), and PBMCs (*n*=34) from controls (*n*=11) and patients (CD, *n*=13; UC, *n*=10) (**Table S2**). We included a standardized blood sample from the same healthy individual at seven intervals during the 12**-**month study period as an internal control. The experimental approach is summarized in **Figure 1A**.

To enable systematic comparisons, we merged the data from all primary cohort samples. The collective dataset contained 4.6 million immune cells from the blood and 3.1 million from the intestinal biopsies. We acquired on average 44,567 immune cells from the ileum, 22,301 cells from the colon, and 118,910 cells from the PBMC samples. A higher number of CD45^+^ immune cells were detected in IBD-inflamed biopsies compared to noninflamed controls (**Figure 1B**). To analyze the large dataset, we utilized Hierarchical Stochastic Neighbour Embedding (HSNE) (20), which at the overview level revealed the global cellular heterogeneity of the dataset within the six major immune lineages (**Figure 1C**).

The cell frequencies of the major lineages in blood from controls and patients were quite similar (**Figure S1B**), except for reduced B cells in UC. In contrast, the ileum and colon samples from patients and controls were distinct (**Figures S1C, D**). For example, a high variation was present in the number of ileal CD8^+^ T cells among controls (range 38.9%-74.3%) and consistently decreased in CD-inflamed biopsies (**Figure S1C**), whereas the ileal CD4^+^ T cells were enriched in CD inflammation. Also, high numbers of B cells were present in the ileum and colon of several patients, and a pronounced abundance of myeloid cells was observed in several inflamed intestinal samples (**Figure S1D**).

Next, we used HSNE to cluster the cells into 343 global partitions and determined the immune composition of each sample. We visualized the clustering of these samples by applying t**-**SNE on cell frequency values. Here, the internal control samples all clustered together (**Figure 1D**, green arrow), demonstrating the reproducibility of the data acquisition between seven batches. Notably, the PBMC and intestinal samples formed two distinct clusters (**Figure 1D**), emphasizing the distinctness of immune phenotypes in blood and intestine. When comparing the immune cell composition of intestinal biopsies from different individuals, considerable interindividual variation was observed (**Figure 1D**). In contrast, multiple samples derived from the same individual but different anatomical locations clustered together, suggestive of a relatively stable individual-specific immune composition in the intestine.

### Dissection of the CD4^+^ T Cell Lineage Into Distinct Cell Populations

We next identified major cell populations within the immune lineages using HSNE, which resulted in 9 distinct CD4^+^ T cell clusters in the 99 intestinal biopsies from the primary cohort (**Figure 2A**). Subset 8 represents CD45RO^–^CD45RA^+^CD27^+^ naïve cells, while subset 4 harbors CD127^low^CD25^high^ T regulatory (Treg)-like cells. The other subsets displayed a memory phenotype, which could be further distinguished by differential expression of CD56, CD161, and CD25, among others. We summarized the unique CD4^+^ T cell marker expression profiles (**Figure 2A**, left panel) and analyzed the population distribution by plotting the cell frequencies of the CD4^+^ T cell subsets for all intestinal samples (**Figure 2A**, right panel). This analysis identified the enriched abundance of the HLA-DR^+^CD38^+^ EM CD4^+^ T cell population 3 in a subset of IBD specimens compared to controls (**Figures 2A, B**). A more detailed analysis of the HLA-DR^+^CD38^+^ EM CD4^+^ T cells at the single-cell level revealed heterogeneous expression of multiple surface markers, including CD161 and the activation markers PD-1 and CD25 (**Figure 2C**).

We next analyzed the transcriptome of HLA-DR^+^CD38^+^ T cells by taking advantage of the publicly available single-cell RNA-sequencing (scRNA-seq) dataset on three treatment-naive IBD-inflamed colons (22) (**Figure S2**). In this dataset, we observed enriched expression of HLA class-II genes and *CD38* in T cells from specimens of patients, in line with the mass cytometry data (**Figure 2B**). Furthermore, gene pathway analysis of *HLA-DRB1* ^+^ *CD38* ^+^ compared to *HLA-DRB1* ^–^ *CD38* ^–^ T cells identified genes associated with the cell cycle (*MKI67* and *PCNA*) and MHC-II antigen presentation (*CD74* and *HLA-DP1*) to be enriched for this cell phenotype (**Figures S2A, B**). In addition, genes associated with effector molecules (*GZMB* and *PRF1*), tissue residency/leukocyte migration (*ITGB7* and *ITGAE*), TCR signaling (*ADA* and *CD226*) and inhibitory markers (*LAG3* and *HAVCR2*) were found in *HLA-DRB1* ^+^ *CD38* ^+^ T cells (**Figure S2C**). Finally, T regulatory (Treg) cell-associated gene *FOXP3* was not expressed by *HLA-DRB1* ^+^ *CD38* ^+^ T cells but was detectable in the *HLA-DRB1* ^–^ *CD38* ^–^ T cell counterpart (**Figure S2D**).

Taken together, this analysis identified intestinal HLA-DR^+^CD38^+^ EM CD4^+^ T cells to be associated with IBD, to be tissue-resident, proliferative, and activated, as assessed at both the protein and RNA level.

### Integrated Immune System Analysis in IBD

We next performed a correlation network analysis of all 44 identified cell populations across the major immune lineages from the 99 intestinal biopsies from the primary cohort (**Figure 3**). This analysis revealed the presence of three main networks of immune subsets. In the first network, 11 cell clusters were correlated, of which 10 were significantly [false discovery rate (FDR) of <5%] more abundant in noninflamed-IBD and control specimens compared to inflamed-IBD specimens (**Figure 3A**, network 1). These cell populations include multiple CD8^+^ T cell clusters, such as innate NK-like CD56^+^ EM CD8^+^ T cells, a highly similar innate NK-like CD56^+^ EM CD4^+^ T cell cluster, but also various innate TCRγδ cells and progenitor ILCs [Lin^–^ ILC (28) and int-ILC (18)] (**Figures 3A, B**).

In contrast, a large group of 21 cell clusters was correlated in a more complex second network, of which 11 cluster frequencies (FDR of <5%) were significantly upregulated in IBD-inflamed biopsies (**Figure 3A**, network 2). This network was characterized by adaptive HLA-DR^+^CD38^+^ EM CD4^+^ T cells, Treg-like cells, and activation marker PD-1-positive EM CD8^+^ T cells, but also by innate CD27^+^ TCRγδ cells, NK cells and CD16^+^ neutrophils (**Figures 3A, C**). Although we could not directly phenotype the latter cells as granulocytes, we could impute cell clusters as neutrophils based on additional data from the validation cohort with a CD15 antibody (**Table S1**).

Lastly, nine immune populations clustered together in a third network (**Figure 3A**, network 3), containing T cell clusters with a naive (CD4_9 and CD8_8) or a CD45RO^+^CCR7^+^ central memory phenotype, as well as naive B cells. As these cell types are abundant in blood but not in the intestine (15, 29), they may represent infiltration from blood.

Visualization of the immune cell subsets from networks associated with the absence of inflammation (network 1) and IBD-related inflammation (networks 2 and 3) with t-SNE at single-cell resolution (**Figures 3D, E** and **S3**) further emphasized the distinctiveness of each cell cluster in these networks.

To validate the results, we next analyzed 89 additional samples from 19 patients and 15 controls (**Table S2**). We used a linear discriminant analysis (LDA) classifier (26) to identify the primary cohort-defined cell subsets in the new samples containing a total of 4.1 million intestinal immune cells. The LDA model efficiently reproduced the cell clusters as defined in the primary cohort, as evidenced with a Pearson R correlation of >0.97 between the true and predicted cell cluster frequencies (**Figure S4**). Comparing the cell frequencies between IBD-inflamed and noninflamed control samples, we identified highly similar groups of immune subsets associated with the presence or absence of IBD-related inflammation in the validation cohort as well (**Figure 3A** and **S5**), attesting to the reproducibility of these results.

By analyzing the collective cell frequencies for each network, this revealed, along with the marked underrepresentation of network 1 in IBD-derived intestinal biopsies, the strong enrichment of network 2 in inflamed specimens from a subset of patients (19/42 patients >50% network 2) (**Figure 3F**). In addition, compared to non-IBD controls, the potential blood-derived immune network 3 was also more abundant in specimens from IBD patients (**Figure 3F**).

Thus, we could distinguish robust intestinal immune cell networks consisting of combinations of adaptive and innate immune cell populations associated with IBD-related inflammation in a subset of patients.

### Immune Cell Profiles in IBD Subtypes, Severity and Intestinal Location

Since we observed similar immune cell profiles in the two patient cohorts, we next pooled the intestinal data (7.2 million cells) in an integrated analysis to reveal associations of cell subsets when stratified for IBD subtype, severity and intestinal location.

With the larger sample size, we now observed increased adaptive HLA-DR^+^CD38^+^ CD4^+^ T cells in inflamed biopsies of both CD and UC subgroups compared to controls. Additional adaptive (Treg and PD-1^+^ CD8) and innate (CD27^+^ γδ, NK and neutrophils) immune cell subsets were also increased in inflamed biopsies compared to controls (**Figure 4A**, left panels). However, we did not observe differences between patients with moderate-to-severe IBD and patients with mild IBD (according to Simple Endoscopic Score for CD (17) and endoscopic Mayo score for UC (30) (**Table S2**) (**Figure 4A**, middle panels). Moreover, most immune subsets elevated in IBD-related inflammation were detected in both the colon and ileum (**Figure 4A**, right panels), except for the more colon-specific increase of HLA-DR^+^CD38^+^ CD4^+^ T cells.

While activated T cells were abundant in inflamed biopsies (**Figure 4A**), innate NK-like CD56^+^ CD4^+^ (**Figure 4B**) and CD56^+^ CD8^+^ T cells (not shown) were decreased in noninflamed controls. A similar shift of balance in composition was displayed for the ILC lineage with increased NK cells and decreased precursor int-ILCs, as well as in the myeloid/granulocyte lineage with increased neutrophils and decreased eosinophils (**Figures 4A, B**). The reduction of these potential regulatory immune subsets (innate NK-like T cells, int-ILCS and eosinophils) was observed in both CD and UC, and did not associate with disease severity (**Figure 4B**).

To extend these findings, we performed principal component analysis (PCA) on all 44 cell population frequencies (**Figure 4C**). Intestinal samples from the two independent cohorts were intermixed, suggesting the absence of batch effects. Moreover, controls were separated from patients, while samples from CD and UC clustered closely together. In addition, noninflamed ileum samples clustered separately from noninflamed colon samples, which converges to a more similar immune composition upon intestinal inflammation. Most strikingly, the variation was mainly due to differences between immune cells from IBD-inflamed and noninflamed tissue samples.

Thus, in both the adaptive and innate immune compartments, cell types were present associating with either IBD-inflamed or noninflamed/control biopsies, where the abundance of these cell types in the inflamed tissue context did not differ between IBD subgroups, disease severity and intestinal location.

### Identification of CD4^+^ T Cells in Blood Bearing Resemblance to the IBD-Associated HLA-DR^+^ CD4^+^ T Cells

**Figure 5:**
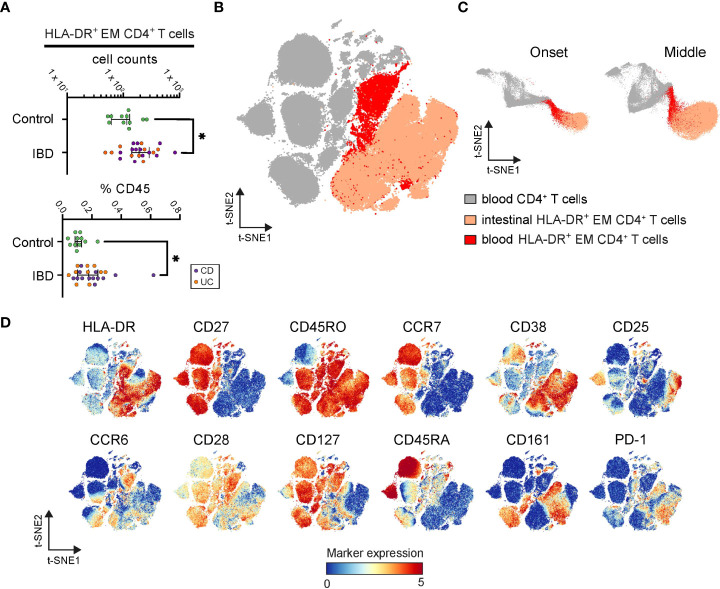
Identification of inflamed instestine-associated CD4^+^ T cells in blood. **(A)** Graphs show frequencies of blood CD4^+^ T cells linked to the intestinal HLA-DR^+^ EM phenotype in each PBMC sample. Bars indicate the median with interquartile range. ^*^p≤0.05, Mann-Whitney test. **(B)** t-SNE embedding of blood CD4^+^ T cells (grey) and classified blood CD4^+^ T cells linked to the intestinal HLA-DR^+^ EM phenotype (red) from 34 PBMC samples, and the HLA-DR^+^ CD4^+^ EM T cells (apricot) from 99 intestine samples. **(C)** t-SNE embedding at the onset and the middle of the computation. **(D)** t-SNE embedding colored for the expression values of the indicated markers.

To determine if disease-associated cell populations are detectable in blood, we applied the LDA classification approach (26). Here only 0.2% of the blood cells matched the phenotype of an intestinal cluster. However, lowering the stringency threshold, by allowing some deviations in the marker expression profiles, identified an HLA-DR^+^ EM CD4^+^ T cell subset in the blood samples that displayed a phenotype similar to one of the most discriminatory cell types in inflamed-IBD biopsies (**Figure 5B**). Notably, a comparison of the frequency of these cells between patients and controls revealed a significantly higher abundance in patients (**Figure 5A**).

Also, these HLA-DR^+^ cells from blood and intestinal tissue were more similar to each other than to other CD4^+^ T cells in the blood, as during the t-SNE-computation (18) (**Figure 5C**) and in the final t-SNE plot (**Figure 5B**), the HLA-DR^+^ cells from blood and intestinal specimens clustered together. In agreement, both blood and intestinal HLA-DR^+^ cells expressed CD45RO and HLA-DR but lacked CD27 and CCR7 (**Figure 5D**). Furthermore, while the blood variant of the HLA-DR^+^ subset expressed higher levels of CCR6, CD28, CD127, and CD45RA, the intestinal variant expressed the tissue-associated/activation marker CD38 and the activation markers CD25, CD161 and PD-1 (**Figure 5D**). Finally, a tiny population in the blood clustered together with HLA-DR^+^ CD4^+^ EM T cells from the inflamed intestine (**Figures 5B, C**), suggesting the presence of an identical phenotype in both anatomical compartments.

### Spatial Colocalization of Identified Immune Subsets in the Intestine

**Figure 6:**
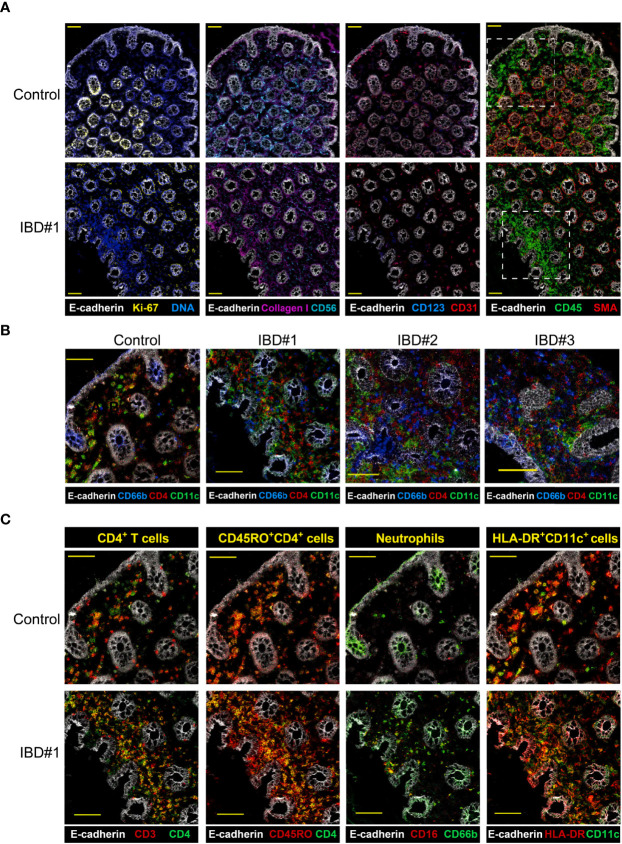
Imaging-mass cytometry reveals immune cell aggregates in the inflamed intestine. Representive images **(A)** structural markers (E-cadherin, Ki67, DNA, Collagen I, CD56, CD31, SMA) and CD45 expression are depicted for IBD biopsies from a control and three IBD patients, yellow scale bar: 100 μm. **(B)** Major immune lineage markers (CD4, CD11c) together with E-cadherin present in a region of a control and three IBD biopsies (dotted line in panel **(A)**, yellow scale bar: 50 μm. **(C)** Co-expresion of CD3 and CD45RO with CD4, CD16 with CD66b, and HLA-DR with CD11c in the same region highlighted in panel **(A)** for a control and an IBD patient biopsy; scale bar: 50 μm.

Finally, to reveal the spatial colocalization of identified cell subsets associated with inflammation, imaging-mass cytometry (IMC) was applied to colon biopsies from 4 treatment-naïve patients with UC and from 4 controls. We designed a 28-antibody panel that incorporated antibodies for structural components, major immune lineages (CD4^+^ T cells, innate lymphoid cells, B cells and myeloid cells), as well as markers indicative of cell differentiation and activation of immune cells (**Table S3**). Visualization of the expression patterns of CD3, CD4, CD7, CD45RO, CD11c, HLA-DR, CD127 and CD161 revealed an even distribution of CD45^+^ immune cells in the biopsies from control subjects, while they accumulated in certain areas of the inflamed IBD biopsies just below the epithelium (**Figures 6A**, **S6.1** and **S6.2**).

Moreover, the analysis of these additional markers identified a dense network of CD4^+^, CD66b^+^ and CD11c^+^ cells within the CD45-rich area in the inflamed samples (**Figures 6B** and **S6.3A**). Colocalization of markers confirmed the presence of memory CD4^+^ T cells (CD3^+^CD4^+^ and CD4^+^CD45RO^+^), neutrophils (CD16^+^CD66b^+^) and HLA-DR^+^CD11c^+^ antigen-presenting cells (APCs) (**Figures 6C** and **S6.3B**). IMC not only allowed us to visualize the interaction between several immune cell subsets in single tissue sections, but also provided a more detailed characterization of the myeloid compartment. For example, we could distinguish conventional dendritic cells (HLA-DR^+^CD1c^+^) and also tissue macrophages (HLA-DR^+^CD163^+^CD68^+^) (**Figures S6.4A, B**).

Thus, we could visualize a dense network of memory CD4^+^ T cells, neutrophils, and several APC types, including conventional dendritic cells and tissue macrophages. Furthermore, the cell phenotypes observed with imaging-mass cytometry closely resembled those found by suspension-mass cytometry

## Discussion

IBD is a chronic inflammatory condition with a remitting and relapsing course. The therapeutic options for IBD are expanding, and therefore the need to develop biomarkers that can predict response to therapies will become increasingly important for personalized medicine decisions. In the present study, we used single-cell mass cytometry to dissect the cellular immune landscape in treatment-naïve patients with IBD in an unbiased manner. We applied this technique to the analysis of fresh intestinal samples, from both inflamed and noninflamed segments, and matched peripheral blood samples of patients and non-diseased controls. This analysis was performed in a primary and a validation cohort and delineated a reproducible network of multiple innate and adaptive immune cell types abundant in 19 out of 42 patients that was associated with inflammation in the intestine and highly distinct from unaffected controls. Moreover, we applied IMC to study the organization of the immune compartment in the tissue context, which showed the colocalization of several immune subsets from the identified cellular network just below the epithelial layer in patients.

Our study identified an increase in the frequency of activated HLA-DR^+^CD38^+^ EM CD4^+^ T cells in treatment-naïve, IBD-inflamed intestinal samples. This is in line with recent studies showing that IBD-related inflammation was associated with memory CD4^+^ T cell responses against the intestinal microbiota (11, 12, 31). We explored an independent publicly-available single-cell RNA-sequencing dataset (22) which revealed abundant *HLA-DRB1^+^CD38^+^* T cells in the inflamed colon of treatment-naive UC patients, thus supporting the relevance of HLA-DR^+^CD38^+^ EM CD4^+^ T cell subset in IBD. Moreover, genes enriched in this phenotype were associated with cell proliferation, tissue residency/leukocyte migration, T cell receptor signaling and cytolytic capacity. Also, in recent studies, HLA-DR^+^CD38^+^ EM T cells were described in the intestinal mucosa of IBD patients (32), and specifically in inflamed Crohn’s disease lesions (33). In these studies, patients were analyzed that were undergoing treatment, as opposed to our treatment-naïve population, thus indicating that these HLA-DR^+^CD38^+^ EM T cells are associated with active inflammation and not greatly affected by the use of medication.

An increase of CD4^+^ T cells with an activated EM phenotype has also recently been described in peripheral blood from adult (34, 35) and pediatric (36) IBD patients. In the latter, these cells also correlated with disease severity. Notably, we identified a higher abundance of HLA-DR^+^ EM CD4^+^ T cells in IBD blood samples compared to controls, cells which strongly resemble HLA-DR^+^CD38^+^ EM CD4^+^ T cells we found in the inflamed intestine. Thus, identifying reliable biomarkers in blood samples would be highly advantageous to circumvent invasive endoscopy, and therefore the monitoring of tissue-specific CD4^+^ T cell responses in blood has substantial clinical potential. Of note, the identified IBD-associated CD4^+^ T cell subset here bears phenotypic similarities to a gliadin-specific subset recently identified in celiac disease and this phenotype was also found in multiple autoimmune diseases (37, 38). This indicates that this disease-associated phenotype can be present on pathogenic antigen-specific CD4^+^ T cells. Moreover, it is well established that human T cells upregulate HLA-class II expression upon antigen-specific stimulation (39–41).

In active tuberculosis, for example, HLA-DR marks a CD4^+^ T cell population that contains recently divided antigen-specific effector T cells (42). Therefore, it is likely that the HLA-DR+ phenotype of the CD38^+^CD4^+^ T cells in IBD similarly reflects recent antigen-stimulation which warrants investigations into the antigen specificity of these cells in patients with IBD in future studies.

By performing a cell subset correlation analysis, we found evidence that the adaptive HLA-DR^+^CD38^+^ EM CD4^+^ cell subset was part of a network of cell subsets that were associated with inflammation in both the primary and validation cohort. This network was further characterized by the presence of Treg-like cells, PD1^+^ EM CD8^+^ T cells, and by innate CD27^+^ TCRγδ cells, NK cells and neutrophils. Of note, since we directly analyzed the intestinal biopsies without cryopreservation, unlike other studies (32, 35), this ensured an accurate interpretation of neutrophils in IBD. Previously, IL-17-producing CD4^+^ T cells (Th17) have been implicated in IBD (43), and it has been shown that they can amplify neutrophil activity (44). *Vice versa*, neutrophils were essential for the induction of Th17 cells (45), and targeting of murine neutrophils led to amelioration of induced colitis (46). Also, IgG- and FcyR-associated intestinal inflammation likely depends on type 17 immunity *via* Th17 cells and TCRγδ cells, which promotes neutrophil and monocyte recruitment (47). Moreover, NK cells can influence CD4^+^ T cell differentiation towards IFNγ-producing cells (Th1) (48). Thus, neutrophils may promote Th17-driven inflammatory responses, while, at the same time, NK cells may promote Th1-driven inflammatory responses, thereby shaping the adaptive immune system in IBD, consistent with the identification of microbiota-reactive CD4^+^ T cells with a mixed Th1/Th17 phenotype (12, 31). Moreover, an expanded CD8^+^ T cell subset expressing PD-1 transcripts that can trigger tissue destruction has recently been identified in UC as well (49).

Our results further imply a role for a network of cell subsets that may influence and/or are dependent on each other, and together underlie inflammation in patients with IBD. Moreover, this network may be further influenced by CD138^+^ plasma cells and stromal cells, as indicated by Martin et al. (33), markers that we had not taken into account in our study. In this respect, it is highly relevant that the crosstalk between myeloid cells and stromal cells has recently been found to be a driver for IBD disease (50) and as a mediator for anti-TNF resistance (51, 52). To determine if key cell subsets in the immune network are colocalized in the inflamed tissue context, we applied imaging-mass cytometry, allowing the simultaneous application of ~40 markers in tissue sections. This analysis revealed a prominent presence of CD11c^+^ myeloid cells in close association with CD66b^+^ neutrophils and CD4^+^ T cells in cellular aggregates just below the intestinal epithelial cell layer in tissue sections from IBD patients. In contrast, these aggregates were not observed in tissue samples of controls. Furthermore, the phenotypes of these IBD-associated cellular aggregates in the tissue slides are consistent with those identified with single-cell mass cytometry analysis.

Strikingly, in our analysis, we could not separate samples from CD and UC patients based on immune cell composition. This may be due to the role of stromal cells in the inflammation, which was not taken into account in the present study. Similarities between CD and UC were previously indicated by the identification of a shared susceptibility allele on chromosome 6 and the observation that both forms of IBD can coexist in a family at a frequency greater than expected (53). Moreover, a recent study characterizing the enhancer and promotor landscape of colon biopsies found that the transcription start sites distinguishing UC and CD were associated with epithelial functions (54). In addition, Kinchen et al. identified a mesenchymal cell population associated with UC (55). Altogether, these findings suggest that while CD and UC share the same immunological features in our study, the pathology may manifest differentially due to differences in the composition and features of the local stromal and epithelial cells. Therefore, for the development of more effective therapeutic approaches, future efforts should be devoted to gain further insights into the interactions between the correlated IBD-associated immune network with non-immune cells.

The work presented here strengthens the concept that inflammation-associated cellular networks can differ between patients. Mechanistic insight into how the distinct cellular components of this network interact and the systematic disruption of these interactions will provide insights for the development of new therapies. This will prove crucial to the development of personalized alternatives for treatment-refractory patients. In conclusion, to develop new approaches to manipulate the immune system in IBD pathology and treat or cure the disease, the next step must be to obtain an understanding of the origin and perpetuation of the disease-associated intestinal immune network in treatment-naive patients.

## Data Availability Statement

The mass cytometry data generated in this study have been deposited at https://flowrepository.org under the identification number FR-FCM-Z55N. In addition, publicly available datasets were analyzed in this study. Sequencing data files were available under the GEO accession number GSE116222. The source code for analyses has been deposited at https://github.com/agneantanaviciute/colonicepithelium.

## Ethics Statement

The studies involving human participants were reviewed and approved by the Medical Ethical Committee of the Leiden University Medical Center (LUMC) (protocol P15.193). All samples were obtained after informed consent, medical ethical commission approval, in accordance with the local ethical guidelines of the LUMC, Erasmus Medical Center and Alrijne hospital, and in accordance with the Declaration of Helsinki. Written informed consent to participate in this study was provided by the participants’ legal guardian/next of kin.

## Author Contributions

VU, LO and FK conceived the study and wrote the manuscript. VU and LO performed most experiments with the help of YK-W, NL, and MS. Also, VU performed most data analyses with the Q16 help of LO, TA, GB, and MP. Moreover, NL, TH, BL, MP and AJ provided conceptual input. AJ, PM, MM, AW, CC, SA, and JE provided clinical material. All authors discussed the results and commented on the manuscript. All authors contributed to the article and approved the submitted version.

## Funding

This work was supported by the Leiden University Medical Center, the Netherlands Organization for Scientific Research [ZonMW 91112008, Rubicon 452181214 to VU], the Crohn’s & Colitis Foundation of America [grant CCFA Ref. 481437], and the collaboration project TIMID [LSHM18057-SGF] financed by the PPP allowance made available by Top Sector Life Sciences & Health to Samenwerkende Gezondheidsfondsen (SGF) to stimulate public-private partnerships and co-financing by health foundations that are part of the SGF.

## Conflict of Interest

The authors declare that the research was conducted in the absence of any commercial or financial relationships that could be construed as a potential conflict of interest.

## Publisher’s Note

All claims expressed in this article are solely those of the authors and do not necessarily represent those of their affiliated organizations, or those of the publisher, the editors and the reviewers. Any product that may be evaluated in this article, or claim that may be made by its manufacturer, is not guaranteed or endorsed by the publisher.

## Abbreviations

CD, Crohn’s disease; EM, effector memory; FDR, false discovery rate; HSNE, hierarchical stochastic neighborhood embedding; IMC, Imaging mass cytometry; IBD, Inflammatory Bowel Disease; ILC, innate lymphoid cell; LDA classifier, linear discriminant analysis classifier; NK cell, natural killer cell; PBMCs, peripheral blood mononuclear cells; sRNA-seq, single-cell RNA-sequencing; tSNE, t-distributed stochastic neighborhood embedding; TH1, T helper 1; Th17, T helper 17; Treg cells, T-regulatory cells; UC, ulcerative colitis.
