# Supplementary material for: Identification of a Disease-Associated Network of Intestinal Immune Cells in Treatment-Naive Inflammatory Bowel Disease
Source: Front Immunol. 2022 Jun 23;13:893803. doi: 10.3389/fimmu.2022.893803 (PMC9260579; doi:10.3389/fimmu.2022.893803)
Supplement: Supplementary file 1 [file DataSheet_1.pdf]

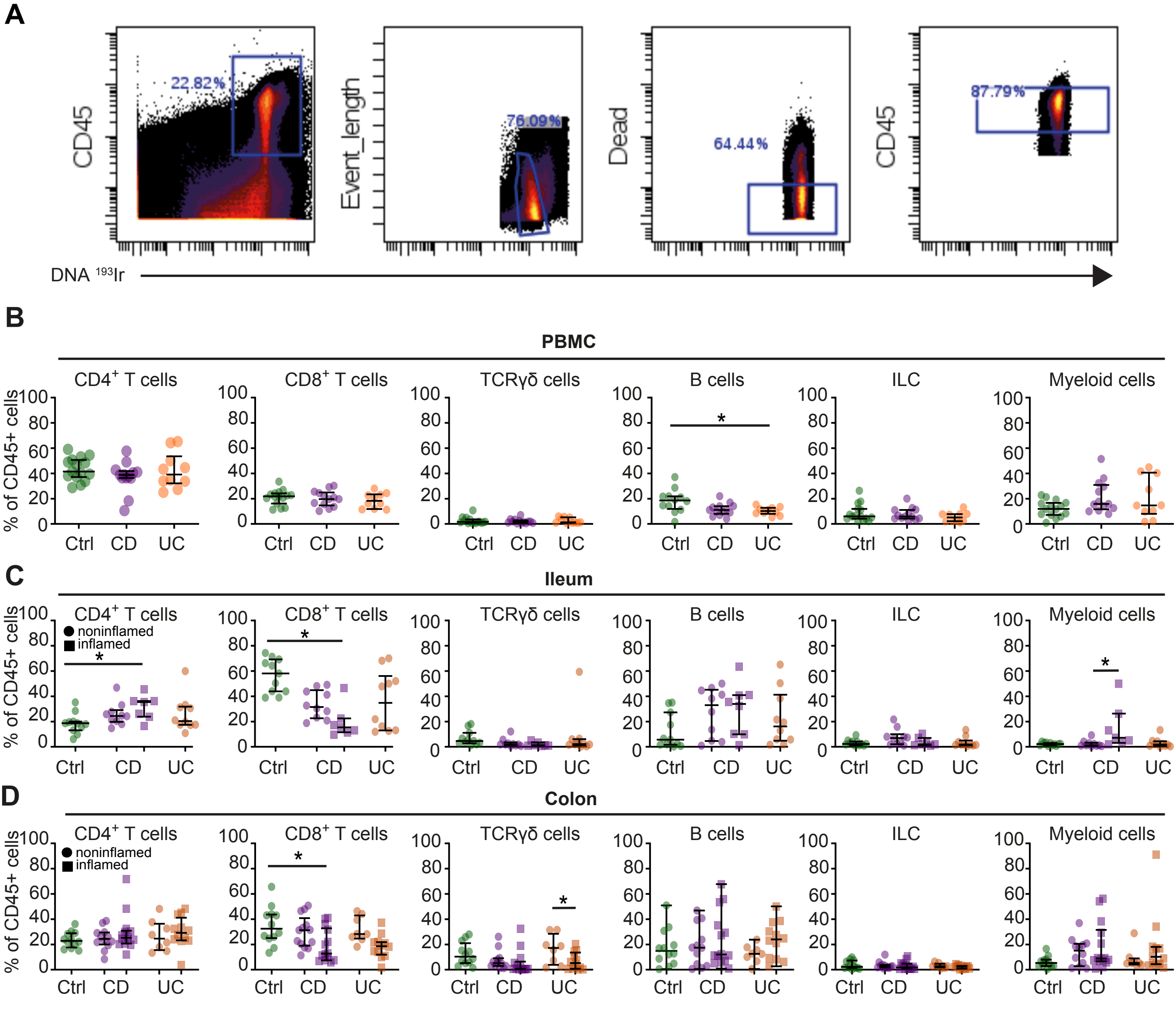

Supplemental Figure 1. Mass cytometry gating strategy and major immune lineage frequencies in PBMC and intestinal samples of IBD patients and controls. (**A**) Mass cytometry gating strategy for single, live $CD45^{+}$ cells of a representative intestinal sample showing sequential gates with percentages. Frequencies (as % of CD45+ cells) of major immune lineages of the primary cohort of (**B**) 34 PBMC samples, (**C**) 41 ileum samples, (**D**) 58 colon samples. Bars indicate median. Each dot represents an individual sample. *P≤0.05, **P≤0.01, Kruskal-Wallis test for multiple groups.

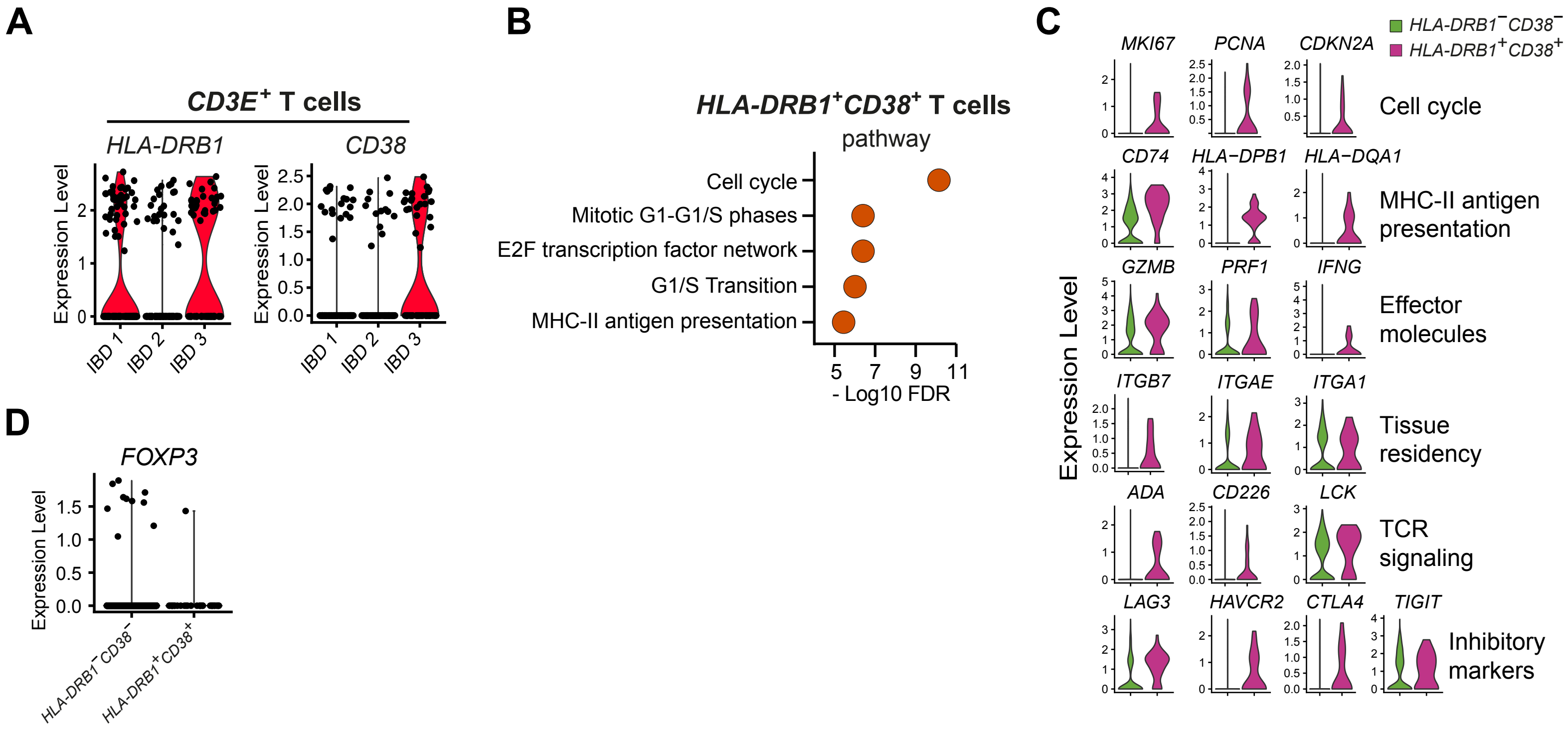

Supplemental Figure 2. Single-cell RNA-sequencing analysis of colon samples from treatment-naive UC patients and controls. (**A**) Violin plots showing expression of genes *HLA-DRB1* and *CD38* on *CD3E*+ T cells from three IBD colons. (**B**) Gene pathway enrichment analysis of upregulated genes by *HLA-DRB1+CD38*+ colonic T cells. (**C**) Violin plots showing expression of multiple upregulated genes by *HLA-DRB1+CD38*+ colonic T cells, and (**D**) expression of *FOXP3*.

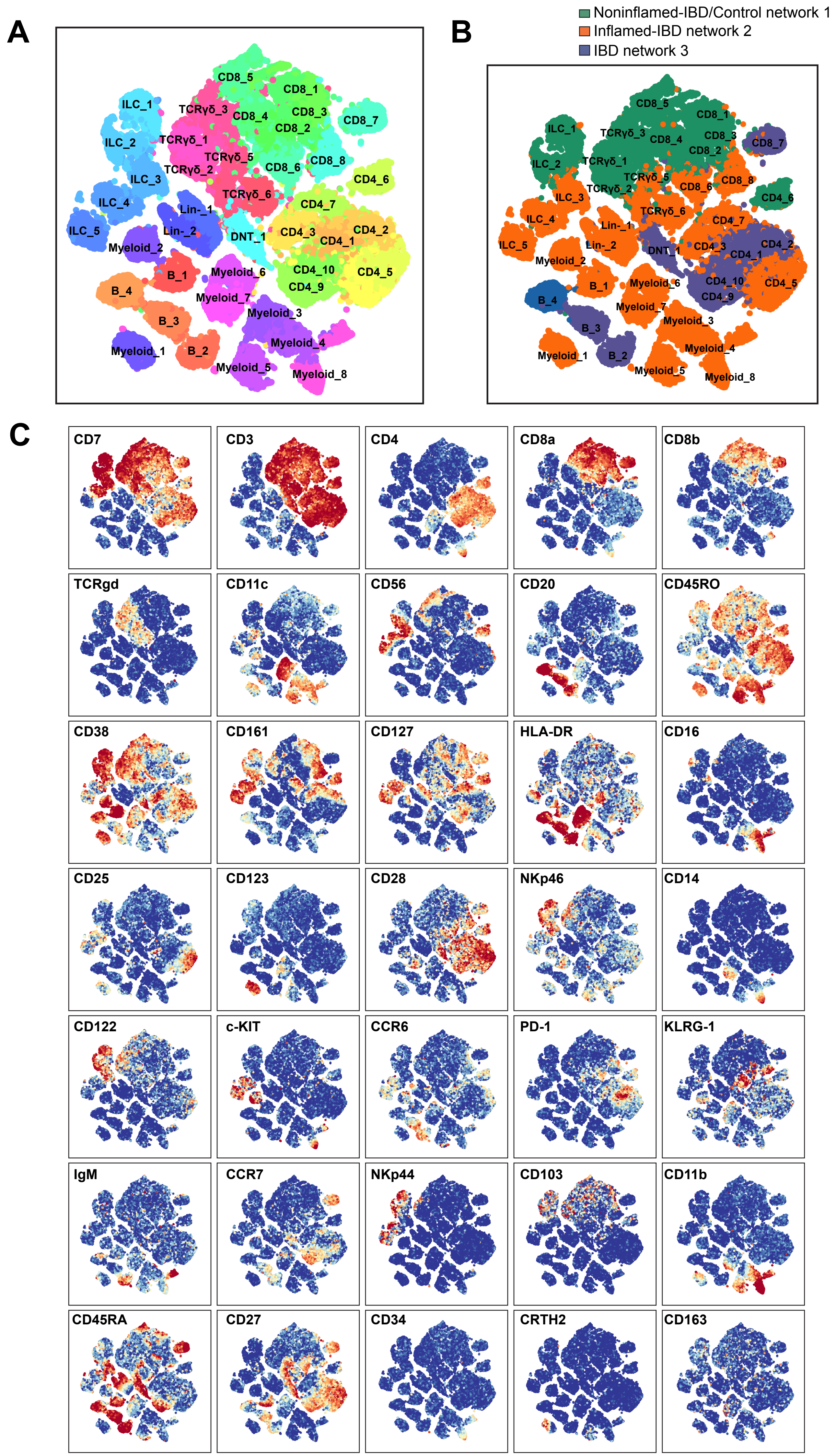
A
CD8_5
CD8_1
ILC_1
TCRγδ_3
CD8_4
CD8_3
CD8_7
ILC_2
TCRγδ_1
CD8_2
TCRγδ_5
CD8_8
TCRγδ_2
CD8_6
ILC_3
CD4_6
TCRγδ_6
ILC_4
Lin-_1
CD4_7
ILC_5
Lin-_2
DNT_1
CD4_3
CD4_2
Myeloid_2
CD4_1
B_1
Myeloid_6
CD4_10
CD4_5
CD4_9
B_4
Myeloid_7
Myeloid_3
B_3
Myeloid_4
Myeloid_1
B_2
Myeloid_5
Myeloid_8
B
Noninflamed-IBD/Control network 1
Inflamed-IBD network 2
IBD network 3
C
CD7
CD3
CD4
CD8a
CD8b
TCRgd
CD11c
CD56
CD20
CD45RO
CD38
CD161
CD127
HLA-DR
CD16
CD25
CD123
CD28
NKp46
CD14
CD122
c-KIT
CCR6
PD-1
KLRG-1
IgM
CCR7
NKp44
CD103
CD11b
CD45RA
CD27
CD34
CRTH2
CD163

Supplemental Figure 3. Networks of intestinal immune cells associated with IBD-related inflammation or controls at single-cell resolution (associated with Figure 3). t-SNE maps of immune subsets (each downsampled to 1,000 cells) from network 1, network 2 and network 3 colored for (**A**) immune subset and (**B**) corresponding network and (**C**) individual marker expressions as indicated.

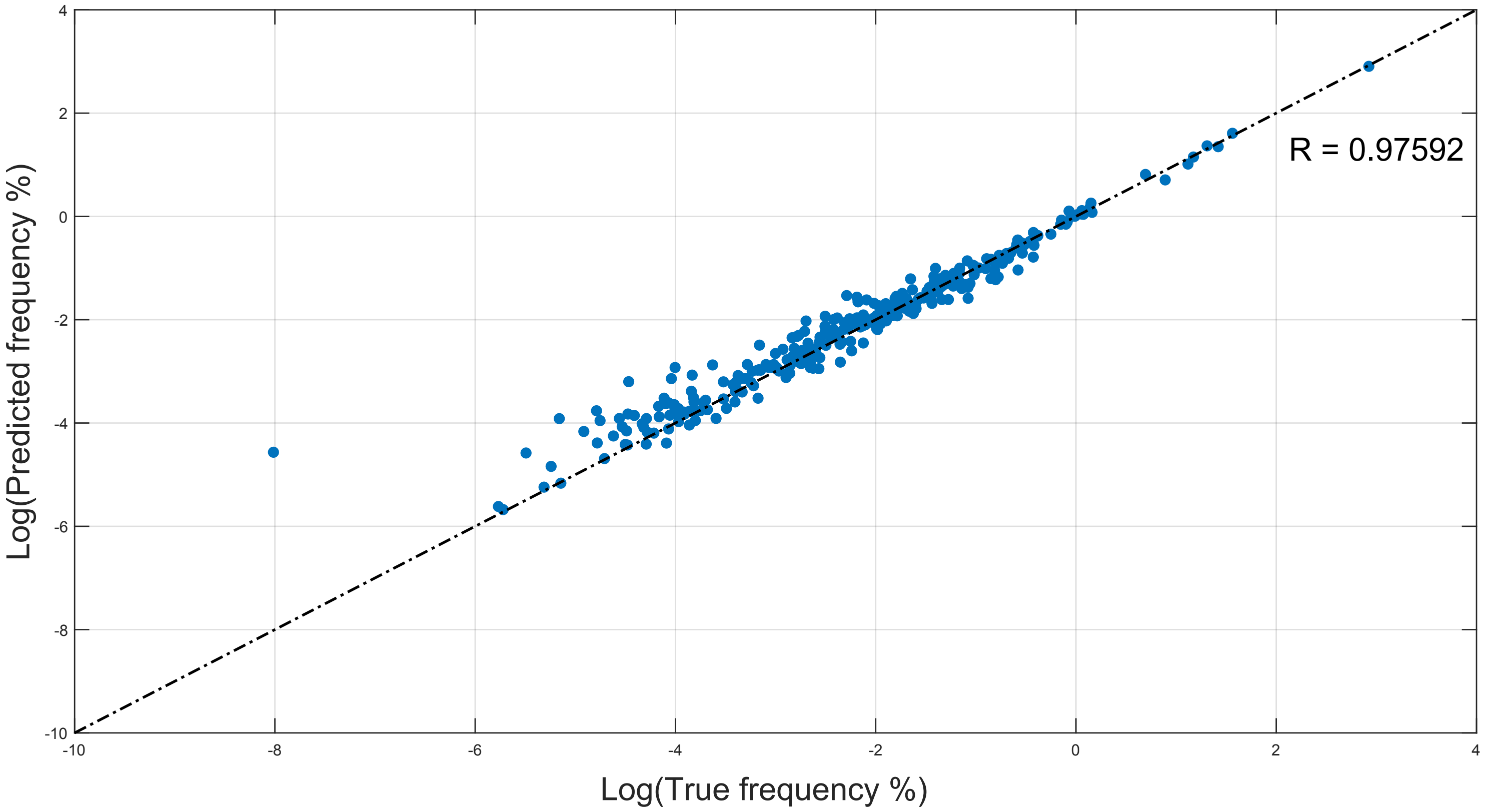

Supplemental Figure 4. Scatter plots between true and predicted cell cluster frequencies. The dashed line shows the least squares fit error line, and the R value represents Pearson correlation coefficient between true and predicted cell cluster frequencies based on the LDA prediction model generated with the primary cohort dataset.

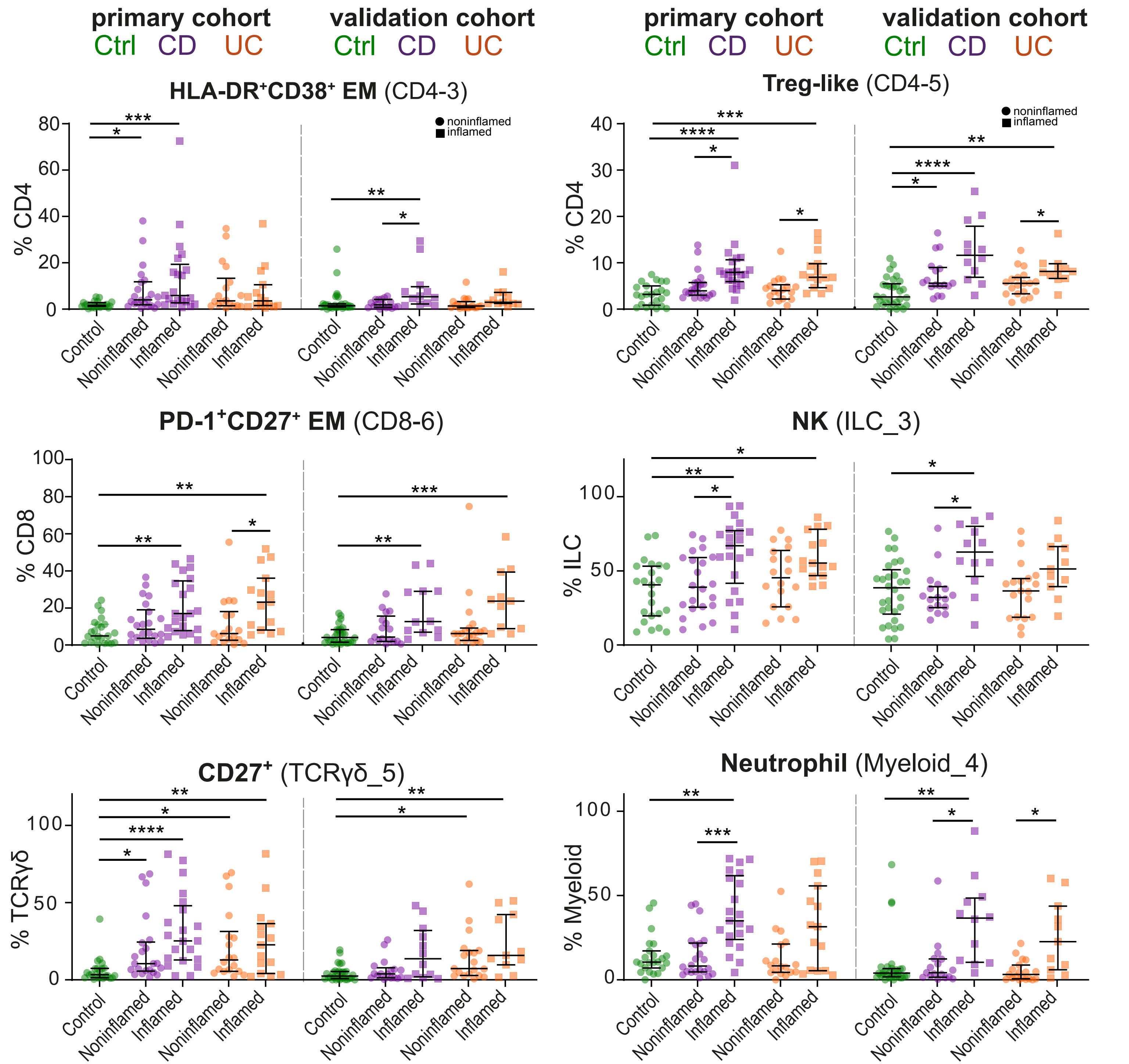

Supplemental Figure 5. Immune subsets primary cohort versus validation cohort. Graphs show frequencies (as % of immune lineage) of indicated subsets from network 2 from primary and validation cohort, stratified for disease subtype (CD; Crohn's disease and UC; ulcerative colitis). Every dot represents an individual intestinal sample. Bars indicate median with interquartile range. *P≤0.05, **P≤0.01, ***P≤0.001, ****P≤0.0001, Kruskal-Wallis test for multiple groups.

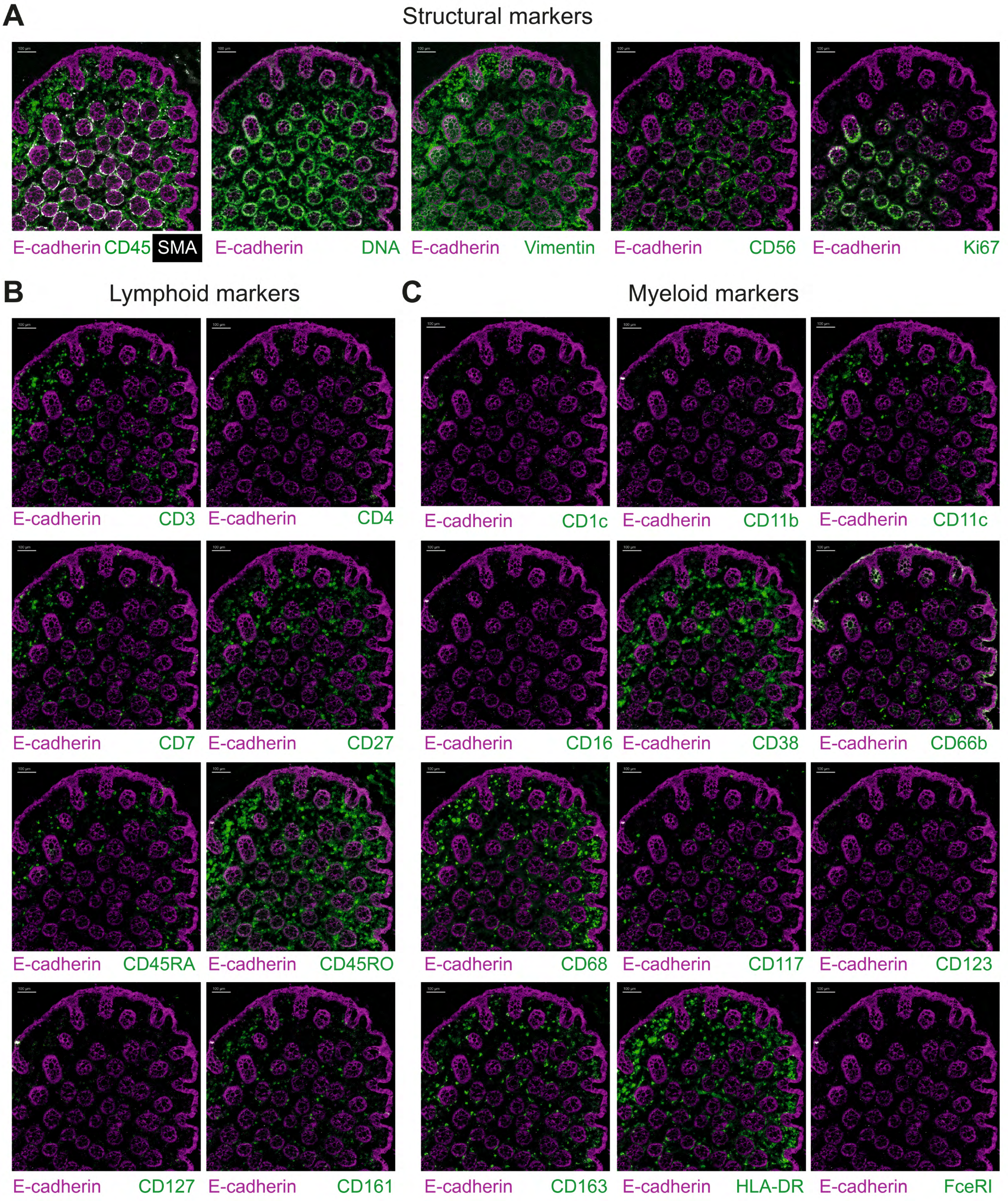

Supplemental Figure 6.1. Panel overview of a representative biopsy from a control individual. Expression of (**A**) structural, (**B**) lymphoid and (**C**) myeloid markers in combination with E-cadherin, detectable with IMC. A representative region of interest (ROI) of 1 mm x 1 mm is depicted.

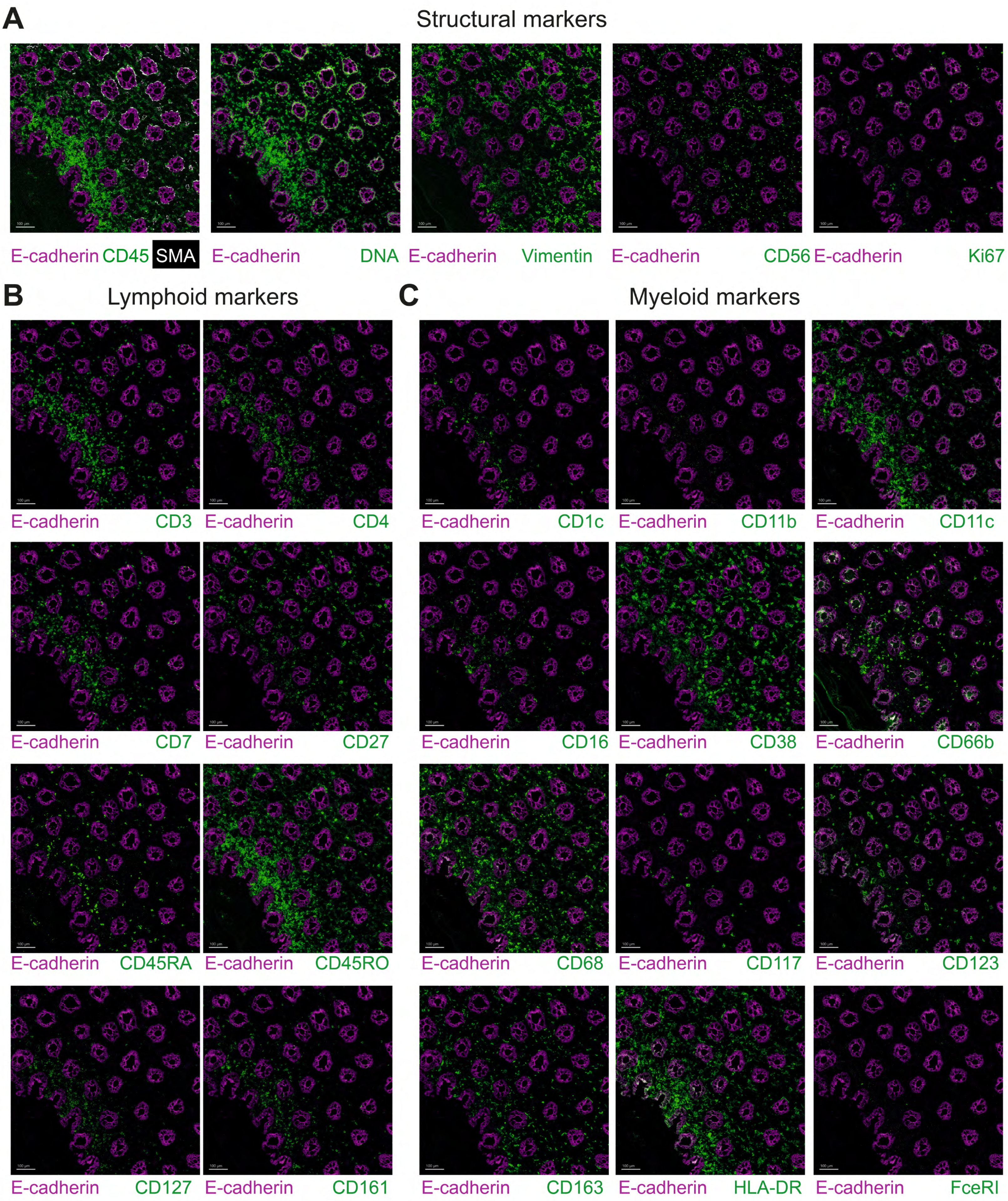

Supplemental Figure 6.2. Panel overview of a representative biopsy of an IBD patient. Expression of (**A**) structural, (**B**) lymphoid and (**C**) myeloid markers in combination with E-cadherin, detectable with IMC. A representative region of interest (ROI) of 1 mm x 1 mm is depicted.

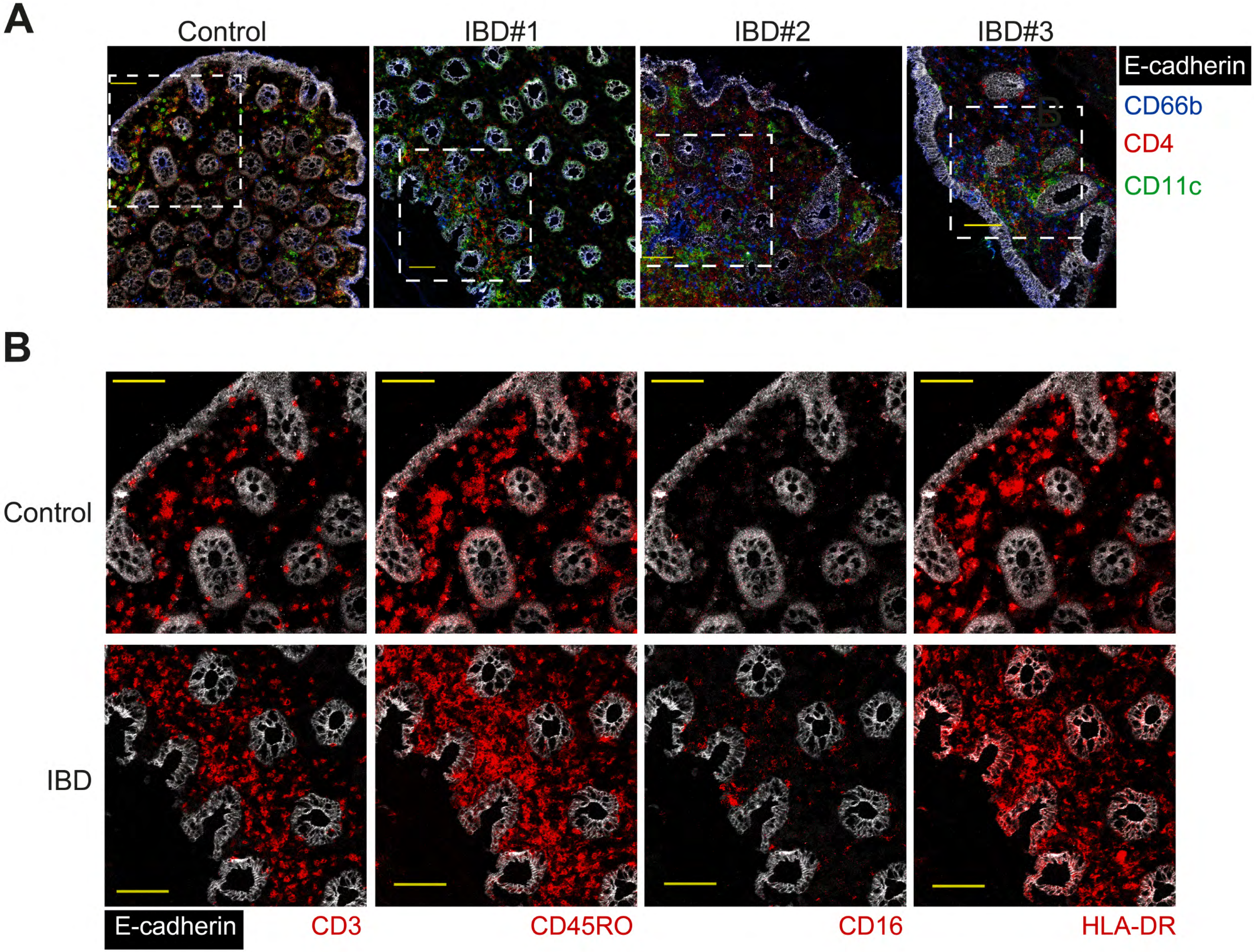

Supplemental Figure 6.3. Colocalization of myeloid cells, granulocytes and CD4⁺ T cells in the inflamed intestine. (**A**) Expression of major lineage markers (CD4, CD66b, CD11c) together with E-cadherin present in the full region of interest (ROI) of 1 mm x 1 mm of a control and three IBD biopsies; scale bar (within dotted region): 50 µm. (**B**) Expression of CD3, CD45RO, CD16 and HLA-DR, together with E-cadherin is shown for a control and three IBD biopsies, scale bar: 50 µm.

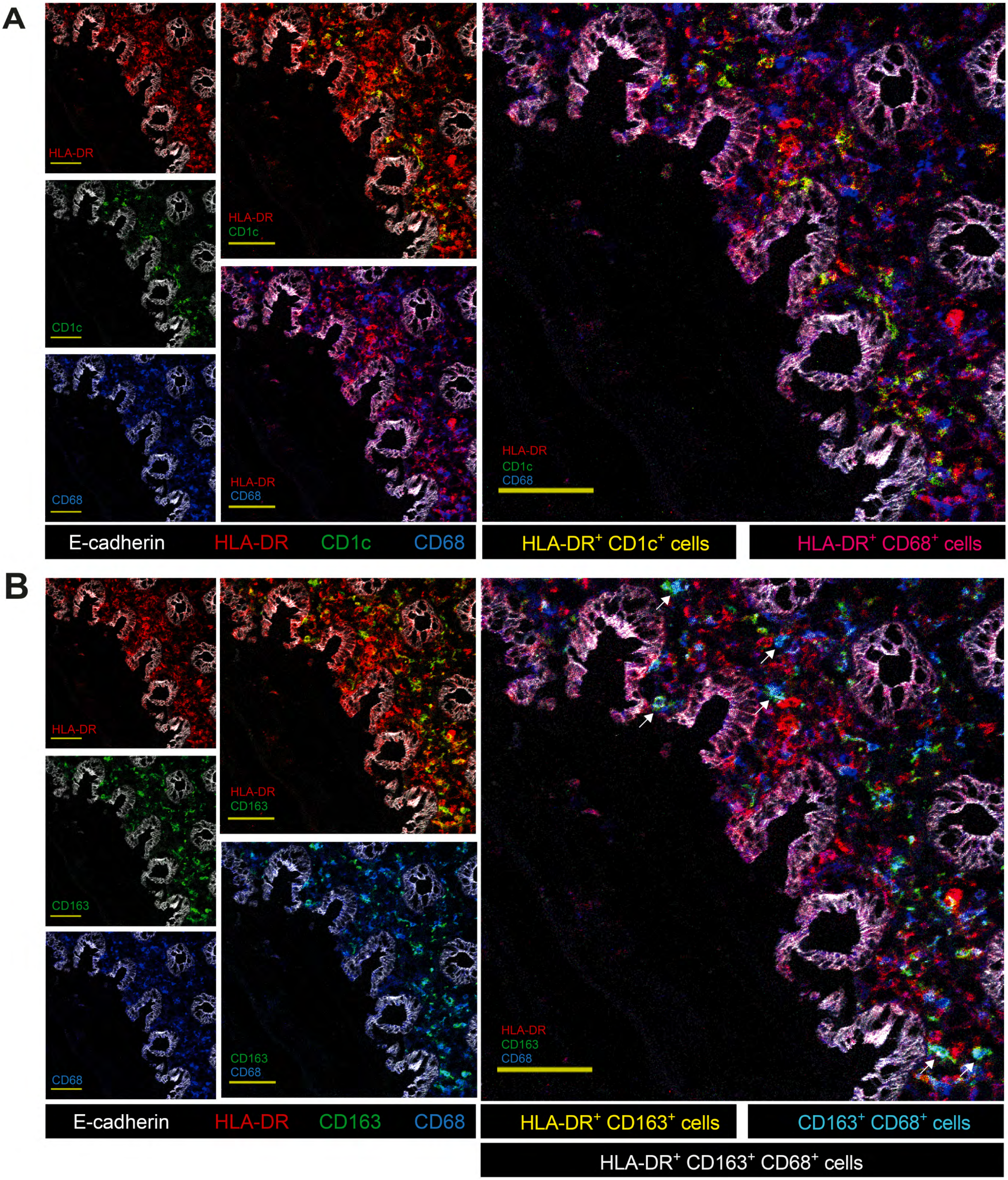

Supplemental Figure 6.4 Characterization of the myeloid compartment in the inflamed intestine. Expression of (**A**) HLA-DR, CD1c and CD68 or (**B**) HLA-DR, CD163 and CD68 together with E-cadherin in the selected region from an IBD biopsy. Expression of single markers is shown in the left panels, combination of two markers in the center panels, overlay of 3 markers is shown in the right panels. Scale bar: 50 µm.

Supplemental Table 1. List of antibodies used for single-cell mass cytometry experiments

	Antigen	Tag	Clone	Supplier	Cat.	Final dilution	Primary cohort	Validation cohort* (a)	Validation cohort** (b)
1	CD127	^{165}Ho	AO19D5	FLM	3165008B	1/200	x	x	x
2	CCR6	^{141}Pr	G034E3	FLM	3141003A	1/100	x	x	x
3	CD8a	^{146}Nd	RPA-T8	FLM	3146001B	1/200	x	x	x
4	CD11c	^{162}Dy	Bu15	FLM	3162005B	1/200	x	x	x
5	CD38	^{172}Yb	HIT2	FLM	3172007B	1/200	x	x	x
6	CD45	^{89}Y	HI30	FLM	3089003B	1/100	x	x	x
7	C-kit	^{143}Nd	104D2	FLM	3143001B	1/100	x	x	x
8	CD4	^{145}Nd	RPA-T4	FLM	3145001B	1/100	x	x	x
9	CD16	^{148}Nd	3G8	FLM	3148004B	1/100	x	x	x
10	CD25	^{149}Sm	2A3	FLM	3149010B	1/100	x	x	x
11	CD123	^{151}Eu	6H6	FLM	3151001B	1/100	x	x	x
12	CD7	^{153}Eu	CD7-6B7	FLM	3153014B	1/100	x	x	x
13	CD163	^{154}Sm	GHI/61	FLM	3154007B	1/100	x	x	
14	CCR7	^{159}Tb	G043H7	FLM	3159003A	1/100	x	x	x
15	CD14	^{160}Gd	M5E2	FLM	3160001B	1/100	x		
16	CD161	^{164}Dy	HP-3G10	FLM	3164009B	1/100	x	x	x
17	CD27	^{167}Er	O323	FLM	3167002B	1/100	x	x	x
18	CD45RA	^{169}Tm	HI100	FLM	3169008B	1/100	x	x	x
19	CD3	^{170}Er	UCHT1	FLM	3170001B	1/100	x	x	x
20	PD-1	^{175}Lu	EH 12.2H7	FLM	3175008B	1/100	x	x	x
21	CD56	^{176}Yb	NCAM16.2	FLM	3176008B	1/100	x	x	x
22	CD11b	^{144}Nd	ICRF44	FLM	3144001B	1/100	x		
23	TCRγδ	^{152}Sm	11F2	FLM	3152008B	1/50	x	x	x
24	HLA-DR	^{168}Er	L243	BioL	307651	1/300	x	x	x
25	CD20	^{163}Dy	2H7	BioL	302343	1/200	x	x	x
26	CD34	^{142}Nd	HIB19	BioL	343531	1/100	x		
27	IgM	^{150}Nd	MHM88	BioL	314527	1/100	x	x	x
28	CD103	^{155}Gd	Ber-ACT8	BioL	350202	1/100	x	x	x
29	CRTH2	^{156}Gd	BM16	BioL	350102	1/100	x	x	
30	CD28	^{171}Yb	CD28.2	BioL	302902	1/100	x	x	x
31	CD45RO	^{173}Yb	UCHL1	BioL	304239	1/100	x	x	x
32	CD122	^{158}Gd	TU27	BioL	339002	1/50	x	x	x
33	KLRG-1	^{161}Dy	REA261	MACS	120-014-229	1/50	x	x	
34	CD8b	^{166}Er	SIDI8BEE	ebio	14-5273	1/50	x	x	x
35	NKp46	^{174}Yb	9E2	BioL	331902	1/40	x	x	x
36	NKp44	^{147}Sm	P44-8	BioL	325102	1/40	x		
37	CD15	^{115}In	W6D3	BioL	323035	1/50		x	x
38	CD14	Qdot800	TüK4	ThermoFisher	Q10064	1/1000		x	x
39	CD1a	^{142}Nd	HI149	Sony	2100510	1/50		x	
40	CD69	^{144}Nd	FN50	FLM	3144018B	1/100		x	x
41	NKp44	^{147}Sm	253415	R&D Systems	MAB22491	1/40		x	x
42	CD5	^{160}Gd	UCHT2	BioL	300627	1/50		x	x
43	CD11b	^{209}Bi	ICRF44	FLM	3209003B	1/100		x	x
44	TIGIT	^{154}Sm	MBSA4	FLM	3154016B	1/100			x
45	CD40	^{142}Nd	5C3	FLM	3142010B	1/100			x
46	PD-L1	^{156}Gd	29E.2A3	FLM	3156026B	1/200			x
47	CD80	^{161}Dy	2D10.4	FLM	3161923B	1/100			x
48	CD57***	^{194}Pt	HCD57	BioL	322325	1/200			x
49	CD66b	^{198}pt	6/40C	BioL	392902	1/40			x

* Panel (a) was used for 9/34 patients in the valdation cohort.
** Panel (b) was used for 25/34 patients in the validation cohort.
*** CD57 was excluded for further analysis.
In grey, the core of markers consistently measured in samples from all patients included.
Fluidigm (FLM), eBioscience (eBio) and Biolegend (BioL).

Supplemental Table 2. Patient characteristics and overview of clinical samples

	Primary cohort			Validation cohort		
**Characteristics**	**Control** (n=11)	**CD** (n=13)	**UC**[a] (n=10)	**Control** (n=15)	**CD** (n=9)	**UC** (n=10)
**Gender, women, n (%)**	8 (72.7)	5 (38.5)	5 (50)	8 (53.3)	7 (77.8)	3 (30)
**Age, years, median [IQR]**	31 (22.5-37)	20 (16-29)	24.5 (20.3 - 33.5)	30 (21.5-36.5)	26 (22-36)	15.5 (14-21)
**Clinical Center endoscopy, n (%)**						
Academic center	8 (72.7)	13 (100)	8 (80)	10 (66.7)	7 (77.8)	8 (80)
Peripheral hospital	3 (27.3)	0 (0)	2 (20)	5 (33.3)	2 (22.2)	2 (20)
**Smoking behavior at diagnosis, n (%)**						
- non-smoker		7 (53.8)	7 (70)		6 (66.7)	10 (100)
- former smoker		4 (30.8)	1 (10)		1 (11.1)	0
- smoker		2 (15.4)	2 (20)		2 (22.2)	0
**Appendectomy in history, yes, n (%)**		0 (0)	0 (0)		0 (0)	0 (0)
**Family history of IBD, yes, n (%)**		3 (23.1)	1 (10)		0 (0)	3 (30)
**Current active EIM**[b]**, yes, n (%)**		0 (0)	1 (10)		1 (11.1)	0 (0)
**Montreal classification CD, n (%)**						
Location						
- L1		3 (23.1)			5 (55.6)	
- L2		6 (46.2)			3 (33.3)	
- L3		3 (23.1)			1 (11.1)	
- L3 + L4		1 (7.7)			0	
Behavior						
- B1		13 (100)			9 (100)	
- p; perianal disease modifier		3 (23.1)			0 (0)	
**Montreal classification UC, n (%)**						
Extent						
- E1			2 (20)			2 (20)
- E2			2 (20)			4 (40)
- E3			6 (60)			4 (40)
Severity						
- S1			3 (30)			1 (10)
- S2			7 (70)			8 (80)
- S3			0			1 (10)
**Severity score**[c]**, n (%)**						
0 (inactive)	11 (100)	0 (0)	0 (0)	15 (100)	0 (0)	0 (0)
1 (mild)	0 (0)	4 (30.8)	3 (30)	0 (0)	6 (66.7)	1 (10)
2-3 (moderate - severe)	0 (0)	9 (69.2)	7 (70)	0 (0)	3 (33.3)	9 (90)
**Type of biopsy, n**						
Terminal ileum unaffected	11	11	10	15	8	10
Terminal ileum affected[d]	0	6	3	0	7	0
Colon unaffected						
- *right-sided*[e]	5	6	5	2	1	4
- *left-sided*[f]	6	6	3	13	8	4
- *rectum*	1	0	0	0	0	1
Colon affected						
-right-sided	0	9	5	0	3	2
-left-sided	0	5	4	0	1	7
-rectum	0	0	3	0	1	2
Total	23	43	33	30	29	30
**PBMC, n**	15	13	10	15	9	10

a three patients were diagnosed as IBD-U (unclassified), and were included in the UC patients group as they were treated the same way.
b in the primary cohort the UC patient suffered from hydradenitits supurativa. The CD patient in de validation cohort had uveitis.
c the severity of any affected segment was classified as inactive (score 0), mild (score 1), or moderate-severe (score 2-3). For Crohn's disease patients this was according to the SES-CD (inactive (0-2), mild (3-6), or moderate-severe (≥7). UC patients were categporized for severity according to the endoscopic Mayo score.
d biopsies exhibiting IBD-associated inflammation as confirmed by endoscopy and/or pathology report were annotated as 'affected', with an exception for the three ileal inflamed biopsies in the UC group. Two of them had atypical inflammation, not related to IBD. One had backwash ileitis. For this reason, these three biopsies were excluded from further quantification analysis.
e the right-sided colon was defined as the cecum, ascendens and transversum.
f the left-sided colon was defined as the sigmoid, and descendens.
Crohn's disease (CD), ulcerative colitis (UC), age at biopsy time point (age), extraintestinal manifistations (EIM), and peripheral blood mononuclear cells (PBMC)

Supplementary Table 3. List of antibodies used for imaging-mass cytometry experiments

	Antigen	Tag	Clone	Supplier	Cat.	Final dilution
1	CD45	^{89}Y	HI30	FLM	3089003B	1/50
2	FcεRIα	^{143}Nd	AER-37 (CRA-1)	BioL	334602	1/50
3	CD4	^{145}Nd	RPA-T4	FLM	3145001B	1/50
4	CD16	^{148}Nd	3G8	FLM	3148004B	1/50
5	E-Cadherin	^{150}Nd	24 E 10	CST	CST 3195BF	1/50
6	CD123	^{151}Eu	6H6	FLM	3151001B	1/50
7	CD127	^{156}Gd	R34.34	Beckman	18LIQ494	1/50
8	CD11b	^{160}Gd	M1/70	BioL	101201	1/50
9a	CD11c*	^{162}Dy	S-HCL-3	BioL	125602	1/50
9b	CD11c	^{162}Dy	Bu15	FLM	3162005B	1/50
10	CD66b	^{163}Dy	6/40c	BioL	392902,0	1/50
11	CD161	^{164}Dy	HP-3G10	FLM	3164009B	1/50
12	CD117	^{165}Ho	104D2	BioL	313202	1/50
13	CD27	^{167}Er	O323	FLM	3167002B	1/50
14	CD1c	^{171}Yb	L161	BioL	331501	1/50
15	CD45RO	^{173}Yb	UCHL1	BioL	304239	1/50
16	CD56	^{176}Yb	NCAM16.2	Thermo	MA1-06801	1/50
17	Collagen I	^{147}Sm	polyclonal	Millipore	AB758	1/200
18	CD31	^{149}Sm	8 9C2	CST	CST3528BF	1/100
19	CD7	^{153}Eu	CD7-6B7	FLM	3153014B	1/100
20	CD163	^{154}Sm	GHI/61	FLM	3154007B	1/100
21	CD45RA	^{169}Tm	HI100	FLM	3169008B	1/100
22	CD3	^{170}Er	UCHT1	FLM	3170001B	1/100
23	CD38	^{172}Yb	HIT2	FLM	3172007B	1/100
24	SMA	^{194}Pt	1A4	CST	56856BF	1/100
25	Vimentin	^{198}Pt	D21H3	CST	5741BF	1/100
26	CD68	^{159}Tb	KP1 (imaging)	FLM	3159035D	1/200
27	Ki-67	^{166}Er	D3B5	CST	CST 9129BF	1/200
28	HLA-DR	^{168}Er	L243	BIoL	307651	1/800

* Two different clones of CD11c used to increase the intensity of the signal
Fluidigm (FLM), Biolegend (BioL), Cell Signaling Technology (CST), eBioscience™ (eBio), Thermofisher (Thermo)
